# A Revival of Epidemiological Entomology in Senegal

**DOI:** 10.4269/ajtmh.18-0162

**Published:** 2018-03-26

**Authors:** Gerry F. Killeen

**Affiliations:** 1 Environmental Health and Ecological Sciences Thematic Group, Ifakara Health Institute, Ifakara, Morogoro, United Republic of Tanzania;; 2 Department of Vector Biology, Liverpool School of Tropical Medicine, Liverpool, United Kingdom

The term *epidemiological entomology* was first coined by Garrett-Jones over half a century ago^1^ but has been out of fashion for far too long.^2^ In this issue, Sougoufara et al.^3^ illustrate clearly just how insightful such an approach can be when applied to characterizing key properties of a dynamic malaria transmission system before and after the scale-up of vector control with long-lasting insecticidal nets (LLINs) in Dielmo, Senegal. Using simple analytical models first pioneered by Garrett-Jones himself,^4^ these authors illustrate how not all may be as it appears based on direct interpretation of entomological data alone. Allowing for the fact that malaria transmission requires both humans and mosquitoes to meet at the same time and place, they show that insecticidal bed nets failed to provide direct personal protection against more than one-third of inoculation events at the outset. Even more worryingly, they demonstrate that this gap in personal protection is growing as mosquitoes adapt to bed nets being a normal part of their environment.

The role of human behavior and the protective effect of bed nets must be accounted for when determining how much exposure to malaria transmission actually occurs, and where and when it occurs in the presence or absence of a bed net.^5–7^ Unfortunately, principles of these analytical approaches dating back to the Global Malaria Eradication Program era have largely been forgotten. The literature now abounds with misinterpreted entomological data, describing the distribution of mosquitoes caught by human landing catch as a direct representation of where and when malaria transmission occurs and how much malaria people are exposed to. Although a number of authors have described that more than half of all transmissions occur outdoors, often at very high entomological inoculation rates, these direct interpretations of human landing catch data are misleading because human participants in landing catches deliberately behave in two very abnormal ways. First, on average, they spend half of the night indoors and half the night outdoors, with no variation in this distribution from dusk until dawn. Second, they do not use bed nets or any other form of personal protection.^5,6^

The average person needs to sleep for 7–8 hours per night. Africa’s most important malaria vectors are human specialists that have evolved to feed most actively in the middle of the night, when most people are asleep. Although these vectors have historically had no strong preference for feeding indoors or outdoors and have been perfectly happy to attack us wherever they find us, it is the preference of humans for the indoor environment that made it the predominant location where malaria was transmitted before the introduction of bed nets.^5–7^ Most people vastly prefer sleeping indoors at night, primarily to protect themselves against much larger threats than mosquitoes. Before the scale-up of bed nets in recent years, these exceptionally human-adapted vector species predominantly encountered and attacked the people of Africa while they were asleep indoors at night, unprotected, and vulnerable. Contrary to common perception, the major vectors of malaria in Africa have never had any strong preference for feeding indoors, known among entomologists as *endophagy*. Instead, they distinguish themselves from the vast majority of mosquito species by being perfectly capable of entering houses, so they can just as readily feed indoors or outdoors, depending on where the best opportunities are found. It was, therefore, the *endophily* of humans, rather than the *endophagy* of mosquitoes, that caused most historical malaria transmission to occur indoors.

In addition to accounting for the influence of human behavioral preferences for spending different times of the night in different places, Sougoufara et al. also account for the protective effect of bed nets use on exposure levels of village residents who do not deliberately expose themselves through human landing catches, a practice that needs to end sooner or later for obvious reasons.^8,9^ Accounting for actual human behavior, true mean exposure levels for bed net users and entire populations were much lower than unadjusted estimates based on raw data from human landing catches or any other trapping method, such as light traps, which operate outside of a bed net. Also, although outdoor exposure may have accounted for only one-tenth of transmission in many parts of Africa before the introduction of bed nets, that is obviously no longer true. Even assuming no change in vector behavior from historical African norms, approximately half of all remaining biting exposure experienced by bed net users has occurred outdoors.^5–7^ However, the estimates presented by Sougoufara et al. illustrate how these documented historical norms merely provide an approximate baseline for a very limited number of locations. They cannot be relied on as being representative of any other part of Africa today, and future behavioral patterns are even more uncertain. Indeed, their estimate that LLINs only provided direct personal protection against 63% of transmission exposure before scale-up is disappointing, differs from historical norms, and helps explain how residual transmission persists in Dielmo. It is even more alarming that these estimates declined to only 45% after several years of high LLINs use. These findings convincingly illustrate why behavioral interactions between mosquitoes and humans need to be continually monitored on a local basis in every African country. Ideally this needs to be done through nationally representative surveillance platforms, rather than opportunistic snapshots from research projects that typically yield intermittent, inconsistently collected data on limited geographic scales.^2,5,7,10^

The scale of the *biological coverage*^7,11^ gap documented by Sougoufara et al. for LLINs in Dielmo was even greater than most of us would have expected based on previous data from other parts of Africa. Although the authors make a laudable case for continued improvements in LLIN availability and use, it must also be acknowledged that the humble bed net has already delivered as much as any of us could have reasonably expected from any single vector control measure.^6,7^ The necessity for complementary new vector control tools to supplement and even supersede LLINs^12,13^ has never been clearer. Indeed, it is particularly noteworthy that Sougoufara et al. have generated such worrisome estimates from an exceptionally well-characterized African setting that has previously been vaunted as “almost there” with respect to malaria elimination.^14^ The findings of Sougoufara et al., therefore, represent a welcome revival of *epidemiological entomology* as a discipline,^1,2^ which not only informs local, national-scale action in Senegal, but also provides an invaluable example for others grappling with similar issues all across Africa.
